# Down-regulation of α-L-fucosidase 1 expression confers inferior survival for triple-negative breast cancer patients by modulating the glycosylation status of the tumor cell surface

**DOI:** 10.18632/oncotarget.4238

**Published:** 2015-05-22

**Authors:** Tzu-Chun Cheng, Shih-Hsin Tu, Li-Ching Chen, Ming-Yao Chen, Wen-Ye Chen, Yen-Kuang Lin, Chi-Tang Ho, Shyr-Yi Lin, Chih-Hsiung Wu, Yuan-Soon Ho

**Affiliations:** ^1^ Graduate Institute of Medical Sciences, College of Medicine, Taipei Medical University, Taipei, Taiwan; ^2^ Department of Surgery, School of Medicine, College of Medicine, Taipei Medical University, Taipei, Taiwan; ^3^ Division of Gastroenterology, Department of Internal Medicine, Shuang Ho Hospital, Taipei Medical University, Taipei, Taiwan; ^4^ Graduate Institute of Clinical Medicine, College of Medicine, Taipei Medical University, Taipei, Taiwan; ^5^ School of Medical Laboratory Science and Biotechnology, College of Medical Science and Technology, Taipei Medical University, Taipei, Taiwan; ^6^ Biological Statistics and Research Consultation Center, Taipei Medical University, Taipei, Taiwan; ^7^ Department of Food Science, Rutgers University, New Brunswick, New Jersey, USA; ^8^ Department of General Medicine, School of Medicine, College of Medicine, Taipei Medical University, Taipei, Taiwan; ^9^ Division of General Surgery, Department of Surgery, Shuang Ho Hospital, Taipei Medical University, Taipei, Taiwan; ^10^ Department of Laboratory Medicine, Taipei Medical University Hospital, Taipei, Taiwan; ^11^ Comprehensive Cancer Center of Taipei Medical University, Taipei, Taiwan

**Keywords:** α-L-fucosidase, triple-negative breast cancer, overall survival rate, glycosylation, metastasis

## Abstract

α-L-fucosidase 1 (FUCA1) is a lysosomal enzyme that catalyzes the hydrolytic cleavage of the terminal fucose residue in breast cancer cells. FUCA1 mRNA levels were detected by real-time PCR, and there was a greater than 139-fold increase in FUCA1 mRNA expression in breast tumor samples compared with normal breast tissue samples (*P = 0.005, *n* = 236). Higher FUCA1 mRNA expression was preferentially detected in early-stage tumors (stage 0 to 2) compared with advanced-stage tumors (stage 3 to 4) (stage 0-1 versus stage 3, *P = 0.015; stage 0-1 versus stage 4, *P = 0.024). FUCA1 protein levels were higher in advanced-stage tumors concomitant with decreased fucosylated Lewis-x antigen expression, as evidenced using the immunohistochemical staining H-score method (*P < 0.001). Statistical analysis revealed that lower FUCA1 levels significantly predicted an inferior overall survival rate among triple-negative breast cancer (TNBC) patients compared with non-TNBC patients (*P = 0.009). Two stable FUCA1 siRNA knock-down MDA-MB-231 cell lines were established, and the results suggest that transient FUCA inhibition creates a selective pressure that triggers the metastasis of primary tumor cells, as detected by wound healing and invasion assays (*P < 0.01). The results suggest that FUCA1 may be a potential prognostic molecular target for clinical use, especially in TNBC patients.

## INTRODUCTION

Glycosylation drives the specific arrangement of the linkages between oligosaccharides and glycoproteins or glycolipids in mammalian cells. Alpha-L-fucose is a key monosaccharide component that is incorporated on the surface of breast cancer cells; this component contributes to many of the fundamental characteristics of breast tumor progression [1, 2]. Alpha-L-fucose-containing molecules are necessary for key aspects of neoplastic progression, including hematogenous metastasis [3], tumor invasion through extracellular matrices (including basement membranes) [2], and up-regulation of Notch signaling [4, 5], with implications for the epithelial-to-mesenchymal transition and activation of breast cancer stem cells. Breast cancer cells incorporate the simple sugar alpha-L-fucose into glycoproteins and glycolipids that are associated with the transformation to a malignant phenotype [1]. For example, the fucosylated Lewis-x antigen is overexpressed on epithelial cells of various origins, including breast cancer cells [1, 6]. Additionally, elevated expression of the Lewis y antigen, which promotes cancer cell proliferation, has been found in 70-90% of human carcinomas of epithelial origin, including breast cancer [7]. Fucosylated Lewis-x antigen expression is a unique prognostic factor for recurrence-free survival and overall survival in younger patients with triple-negative breast cancer (TNBC) [6, 8]. Specific targeting of these proteins with a radiolabeled humanized monoclonal antibody significantly inhibited tumor growth in an MCF-7 breast cancer xenograft model in BALB/c nude mice [9]. These results suggest that fucosylated glycoconjugates on the breast cancer cell surface can be used as therapeutic molecules [2, 10, 11].

Most cases of breast cancer originate in the lactiferous ducts and do not spread beyond the milk duct into normal surrounding breast tissue; this type of breast cancer is defined as non-invasive breast cancer (ductal carcinoma in situ, DCIS). A previous study suggested that lysosomal enzymes are secreted into the lactiferous ducts; then, these hydrolytic enzymes damage cells, leading to the initiation of breast cancer [12]. In the present study, we demonstrated that the lysosomal enzyme, α-L-fucosidase 1 (FUCA1, EC number 3.2.1.51) that catalyzes the hydrolytic cleavage of terminal fucose residues was preferentially detected in early-stage (stage 0 to 2) breast cancer tissues (*n* = 236, *P = 0.015 and 0.024, respectively). This result suggests that FUCA-mediated decreases in the composition and quantity of cell surface fucosylation-associated molecules could critically reduce the invasiveness of cancer cells in early-stage breast cancer. FUCA has also been studied because of its potential utility in the clinical diagnosis of hepatocellular carcinoma [13, 14] and colorectal cancer [15]. Another study demonstrated that FUCA in combination with CD26 represented a molecular diagnostic marker, especially for non-disseminated colorectal cancer [16]. All of these studies reported that FUCA is preferentially detected during the early stages of cancer development. However, the mechanism by which FUCA is involved in breast cancer progression is not fully understood.

Secreted FUCA has been identified as the key enzyme responsible for the defucosylation of terminal epitopes. For example, a previous study demonstrated that L-fucose was transferred from the surface of human gastric cancer cells to a co-cultured clinical strain of *H. pylori* [17]. Another study demonstrated that FUCA pretreatment significantly decreased the invasive capability of MDA-MB-231 breast cancer cells [2]; this effect was reversed by deoxyfuconojirimycin, a specific FUCA inhibitor. Because α-L-fucose-containing molecules are readily detected on migratory cancer cells, there is a rationale for studying the potential ability of FUCA to modify fucose expression on breast tumor cells. FUCA can remove α-L-fucose from oligosaccharide sites on highly invasive and metastatic breast cancer cells. Therefore, we hypothesized that high FUCA expression could decrease the expression of fucose-containing molecules on the surface of cancer cells, thereby significantly inhibiting tumor cell invasion.

In this study, we evaluated FUCA1 expression in breast cancer tissue samples from patients with different stage disease. Lower FUCA1 expression was preferentially detected in tissues from patients with advanced-stage (stage 3 to 4) breast cancer. TNBC patients often face a high risk of early relapse that is characterized by extensive metastasis. A recent study using lectin microarrays determined that the binding of TNBC cells to Ricinus communis agglutinin I was proportional to their metastatic capacity [18]. They also found that this binding inhibited cellular invasion, migration, and adhesion; a membrane glycoprotein, POTE ankyrin domain family member F, was identified that may play a key role in mediating these effects [18]. Previous studies have shown that aberrant cell surface glycosylation is associated with cancer metastasis, suggesting that altered glycosylation might be a diagnostic indicator of metastatic potential [19]. To strengthen our hypothesis that FUCA1 is a biomarker for poor prognosis, we analyzed the correlation between FUCA1 mRNA expression and disease state and found that lower FUCA1 mRNA levels significantly predicted inferior overall survival for TNBC patients (*P = 0.009). Our results suggest that FUCA1 is an indicator of poor prognosis for patients with advanced-stage TNBC.

## RESULTS

### FUCA1 mRNA is more highly expressed in human breast tumor tissues

**Figure 1:**
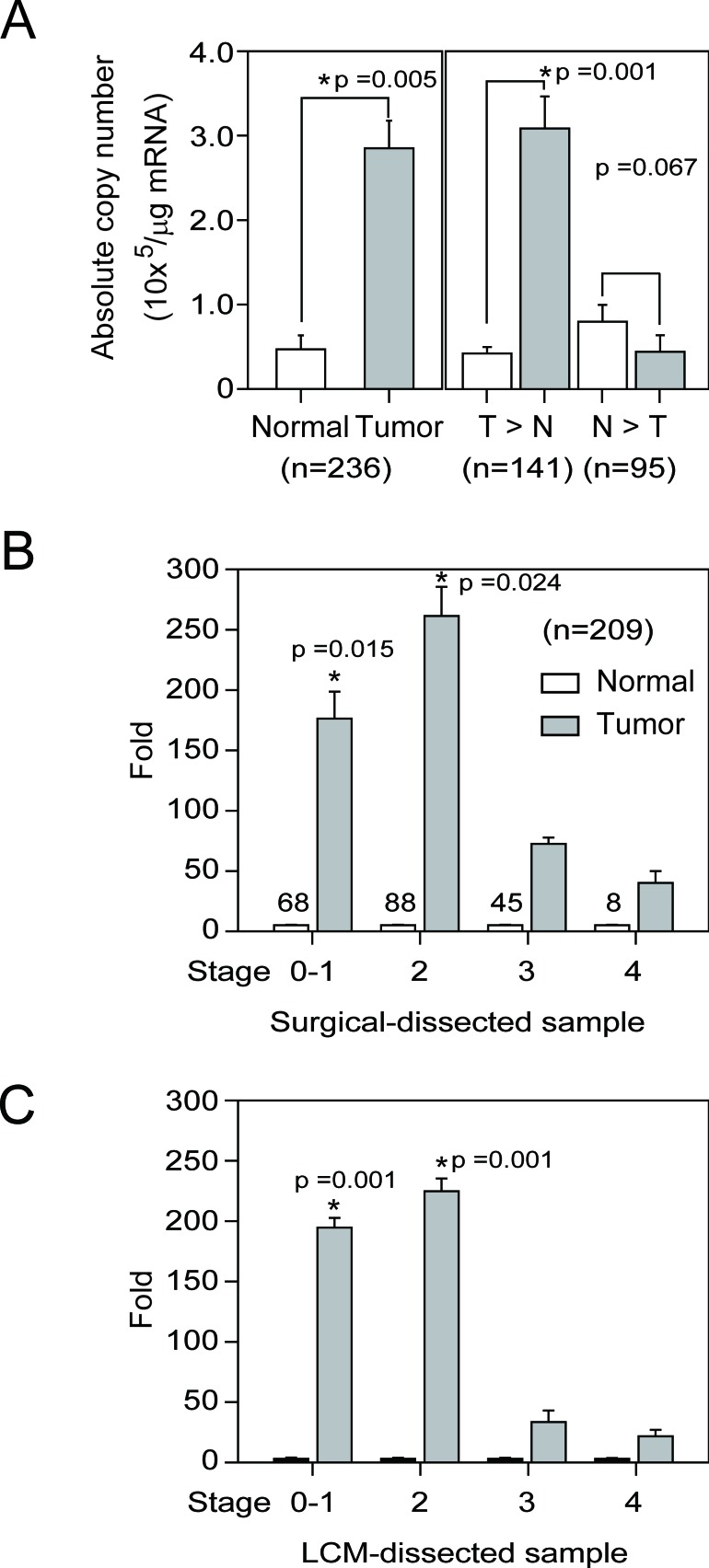
FUCA1 mRNA expression levels in normal and malignant human breast tissues **A.** FUCA1 mRNA expression profiles in paired human breast tumor (red lines) and normal (green lines) tissues (*n* = 236) were evaluated by real-time PCR. **B.** FUCA1 mRNA expression levels in 141 patient samples with higher expression in tumor tissue compared with normal tissue (T > N) and in 95 samples with higher expression in normal tissue compared with tumor tissue (N > T). Copy numbers (× 10^3^ per μg mRNA) were calculated from the mean real-time PCR data; error bars indicate the 95% confidence interval (C.I.). Tumor *vs*. normal tissue in group 1 (T > N), *P* = 0.001; normal *vs*. tumor tissue in group 2 (N > T), *P* = 0.067. The data were analyzed using a paired *t*-test with two-sided *P-*values. **C.** Left panel: Tumor-normal tissue pairs were divided into four subgroups according to clinical staging criteria recommended by the American Joint Committee on Cancer (AJCC). The data are presented as the mean of the fold change in expression for paired tumor and normal tissues. Right panel: Tumor-normal tissue pair samples were analyzed using LCM to establish the relative expression of FUCA1 mRNA expression [33]. Error bars indicate the 95% C.I. The numbers of paired samples at each stage are indicated above the bars. The data were analyzed with an overall nonparametric test (Kruskal Wallis test), and multiple comparisons were assessed with the Mann-Whitney test. The following comparisons were performed: stage 0-1 *vs*. stage 4, *P* = 0.0015; and stage 2 *vs*. stage 4, *P* = 0.024. All the *P*-values are two-sided.

FUCA1 mRNA levels were examined in paired tumor and normal tissue samples by real-time RT-PCR analysis (*n* = 236). The average FUCA1 mRNA (copy number x 10^3^/μg) expression was 139-fold higher in tumor tissue than in normal cells (Figure 1A, bars 1 *vs*. 2, *P = 0.005, *n* = 236). The cases were further divided into two groups according to FUCA1 mRNA expression. Nearly 60% (*n* = 141) of the cases fell into Group 1 (tumor > normal, T > N); in this group, the mean FUCA1 expression level in the tumor samples was 148-fold greater than that in the normal samples (Figure 1A, bars 3 *vs*. 4, *P = 0.001). Within Group 1, higher FUCA1 expression (defined as > 100-fold) was detected in 58% (82/141) of the tumor tissue samples (data not shown). However, in Group 2 (normal > tumor, N > T), the FUCA1 expression level in 72% (69/95) of the normal tissues was less than 20-fold greater than that in the tumor tissues (Figure 1A, bars 5 *vs*. 6).

### FUCA1 protein expression is higher in early-stage breast tumor tissues

On average, elevated FUCA1 mRNA expression levels were significantly associated with early-stage tumors rather than advanced-stage tumors (Figure 1B, stages 0-1 and 2 *vs*. stage 4, *P = 0.015 and stage 2 *vs*. stage 4, *P = 0.024, respectively). To confirm these observations, tumor and normal cells were harvested separately by laser capture microdissection (LCM) from 12 tumor samples (*n* = 3 for each group); FUCA1 mRNA expression in the LCM cells was determined by real-time PCR (Figure 1C). Increased FUCA1 mRNA expression was preferentially detected in tissue samples from patients with early-stage disease (Figure 1C; T/N ratio: stage 1 = 191-fold (*P = 0.001 *vs*. stage 4), stage 2 = 230-fold (*P = 0.001 *vs*. stage 4), stage 3 = 35-fold (*P = 0.06 *vs*. stage 4), and stage 4 = 23-fold).

**Figure 2:**
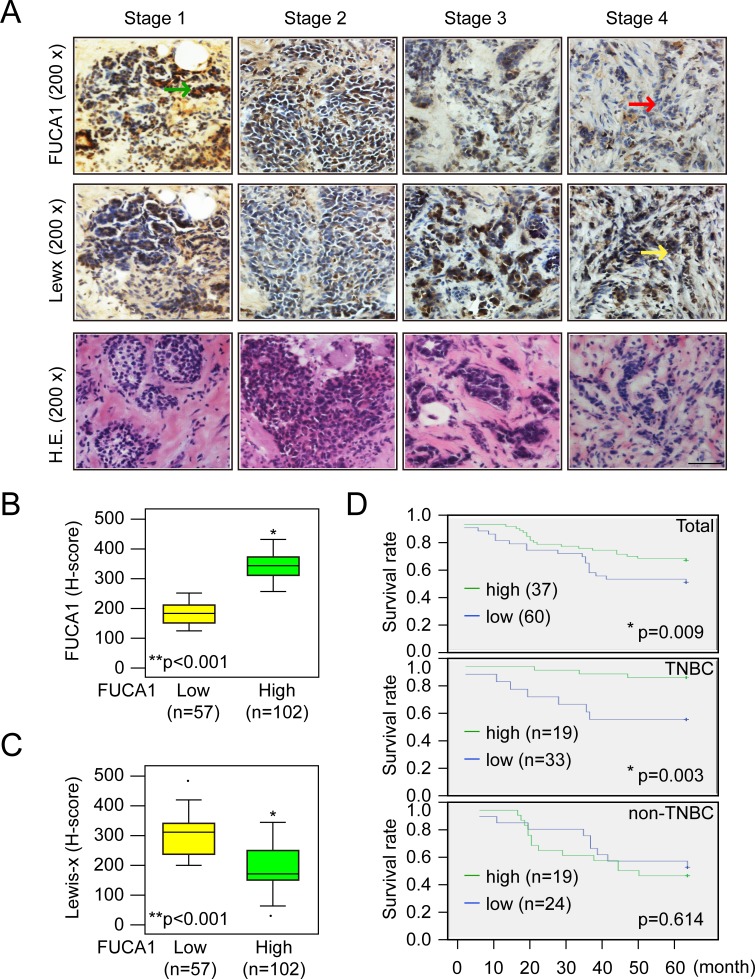
Higher FUCA1 expression predicts better survival of breast cancer patients **A.** Immunolocalization of FUCA1 and Lewis-x antigens in different stages of human breast cancer. The tumor tissues were cut into 5-μm serial sections and stained with antibodies specific to human FUCA1 or fucosylated Lewis-x antigen. H.E., hematoxylin and eosin stain. Scale bar = 200 μm. **B.**, **C.** Histological and quantitative analysis of FUCA1 and Lewis-x antigen expression in breast cancer patients (**P < 0.001; (2-tailed) Pearson correlation test). **D.** Kaplan-Meier analysis of the overall survival of 159 breast cancer patients with low or high FUCA1 H-scores.

FUCA1 protein expression was determined by IHC staining of frozen tumor sections. The results revealed increased FUCA1 protein expression in premalignant DCIS (Figure 2A, indicated by a green arrow). In contrast, lower FUCA1 expression levels were detected in advanced-stage (stage 4) breast cancers (Figure 2A, indicated by a red arrow). The clinical status of each patient was determined to ascertain whether higher FUCA1 expression in early-stage tumors was important for clinical/therapeutic outcomes (Table 1). Our results indicate that higher FUCA1 expression (T > N) was positively correlated with nodal status and tumor stage (*P = 0.004 and *P = 0.004, respectively). High FUCA1 expression in tumor tissue (T > N) was also associated with the non-smoking status in these patients (*P = 0.037).

**Table 1:** Demographic evaluation of clinical criteria and changes in FUCA1 mRNA expression fold ratios of tumor/normal paired samples

Factors	FUCA1 N>T			FUCA1 T>N		
	(n)	§mean (95%CI)	*P* Value	(n)	§mean (95%CI)	*P* Value
Age			0.218			0.286
<54yr	39	59.6 (−33-145)		113	151 (−128.4-38)	
≧54yr	29	3.97 (−22-133)		111	197 (128.5-38)	
Size of tumor			0.701			0.395
Tis	6	10.6 (−16-37)		12	291 (−35-618)	
T1	31	68.8 (−29-166)		75	168 (101-234)	
T2	25	0.45 (0.32-0.58)		101	182 (119-245)	
T3	3	0.44 (−0.58-1.47)		31	156 (46-267)	
T4	4	57.9 (−124-240)		8	7.6 (−2.1-17)	
Nodal status			0.49			0.004*
N0	10	86.9 (−65-239)		85	188 (123-252)	
N1	15	0.31 (0.14-0.48)		86	267 (166-368)	
N2	22	27 (−11.7-65.8)		40	55 (24-87)	
N3	12	8.68 (−8.3-79)		34	104 (15-193)	
Stage			0.329			0.004*
Tis	20	29.6 (−13-72.4)		18	53.5 (17-89)	
I	12	133 (−130-397)		50	214 (120-308)	
II	17	13.9 (−14-42.6)		88	264 (183-345)	
III	10	0.47 (0.18-0.74)		45	77 (10-143)	
IV	0			8	39 (−31-110)	
Grade differentiation			0.963			0.136
poorly	12	33 (−29-95)		36	205 (67-343)	
moderate	41	44 (−28-116)		120	134 (92-176)	
high	12	19 (−22-62)		56	247 (140-353)	
Smoke status			0.707			*0.037
Non-	29	60 (−42-163)		128	226 (162-291)	
Current-	12	52 (−20-125)		19	110 (10.8-209)	
Passive-	14	0.36 (0.18-0.54)		30	92.8 (39-146)	
Even-	12	5.5 (−5.8-16.9)		15	46.8 (18-75)	
ER status			0.38			0.64
Negative	25	9.64 (−131-50.7)		87	161.7 (−105-65)	
Positive	44	50 (−111-30.2)		139	181 (−105-61)	
PR status			0.43			0.46
Negative	34	17.7 (−122.38-52.8)		118	190 (−52-115)	
Positive	35	52.6 (−122-52.98)		106	158 (−51-114)	
Her-2 status			0.311			0.385
Negative	41	17.1 (−154-50)		140	192 (−52-134)	
Positive	21	69.3 (−197-93)		68	151 (−49-132)	
Chemotherapy			0.402			0.583
Non-treatment	21	13.7 (−10.7-38.2)		36	130 (57-203)	
Post-treatment	33	65 (−26-156)		99	172 (109-234)	
Pre-& Post-treatment	0			5	385 (−37-114)	
ND	17	0.41 (0.24-0.58)		91	193 (120-266)	
Radiotherapy			0.559			0.17
Non-treatment	38	54.9 (−23-133)		101	184 (125-243)	
Post treatment	16	21.7 (−23-67)		39	855 (8.65-162)	
ND	17	0.41 (0.24-0.58)		91	193 (120-266)	
Tamoxifen			0.29			0.677
Non-treatment	31	17.7 (−4-39.6)		71	151 (90-212)	
Post treatment	23	82 (−50-214)		69	162 (86-237)	
ND	17	0.41 (0.24-0.58)		91	193 (120-266)	
Herceptin			0.006			0.311
Non-treatment	46	20 (−0.48-40.6)		101	137 (89-184)	
Post treatment	6	252 (−364-868)		17	243 (18.5-468)	
Pre- & Post-treatment				3	8.75 (−22.4-39.9)	
ND	19	0.38 (0.23-0.54)		110	195 (129-261)	

Fold ratios of FUCA1 mRNA expression were determined in normal/tumor or tumor/normal paired samples. Data were analyzed using either the Independent T-test (ER, PR, Her-2, and Age groups) or by One-Way ANOVA analyses for the other groups.

* A P-value < 0.05 was considered as statistically significant. All P-values are two-sided.

§ mean: average fold ratio of FUCA1 mRNA expression in each group.

A previous study demonstrated that increased fucosylation of Lewis-x antigens by 5 fucosyl-transferases (FUT-3, -4, -5, -6, and -7) was detected in breast cancer cells that preferentially metastasized to the bone [20]. Moreover, fucosylated Lewis-x antigens are a poor prognostic factor in younger patients with TNBC [1, 6, 8]. Because FUCA1 catalyzes the hydrolytic cleavage of the terminal alpha-L-fucose residue in glycoproteins and glycolipids that are associated with the transformation to a malignant phenotype [1], we quantified the levels of fucosylated Lewis-x antigens in breast tumor tissues with high or low FUCA1 expression using IHC H-score staining assays, as described in the Materials and Methods section (Figure 2A, 2B and 2C). FUCA1 was preferentially detected in early-stage breast cancer tissues (Table 1, Figures 1C and 2A). Interestingly, lower FUCA1 expression was associated with highly fucosylated Lewis-x antigens, such as those detected in the advanced-stage breast cancer tissues (Figure 2A, indicated by a yellow arrow), and vice versa (Figure 2B and 2C, H-score data).

High FUCA1 expression levels can alter the composition and decrease the quantity of cell surface fucosylation-associated molecules, thereby limiting the invasiveness of early-stage (stage 1 and 2) breast cancer cells. Next, we investigated whether low FUCA1 levels were detected in patients with higher levels of fucosylated Lewis antigen. We divided patients into two groups according to their FUCA1 H-scores. The mean FUCA1 H-score in 159 breast cancer patients was 273. Therefore, FUCA1 H-scores < 273 defined the low FUCA1 group (*n* = 57), and vice versa (Figure 2B). In the high FUCA1 group, higher FUCA1 expression was concomitantly detected with lower fucosylated Lewis antigen levels (*P < 0.001) (Figure 2B and 2C). To further strengthen our hypothesis that lower FUCA1 expression is a biomarker of poor prognosis, we analyzed the correlation between FUCA1 protein levels (indicated by H-score) and overall survival (Figure 2D). Lower FUCA1 expression was significantly associated with an inferior overall survival rate in breast cancer patients (Figure 2D, *P = 0.009). We subdivided the patients into TNBC and non-TNBC groups and analyzed the correlation between FUCA1 protein levels (indicated by H-score) and overall survival in each group (Figure 2D). Interestingly, lower FUCA1 protein expression (H-score < 273) was associated with poor overall survival among TNBC patients (Figure 2D, *P = 0.003) but not among non-TNBC patients.

In the statistical analyses, a hazard of 1.000 was defined as the baseline for patients with the following conditions: T4, N3, and stage IV. Cox regression proportional hazard analysis was used to evaluate whether the FUCA1 H-score was a prognostic indicator. The univariate Cox regression revealed that FUCA1 H-scores were associated with the risk of death for TNBC patients (*P = 0.023) but not for non-TNBC patients (Table 2). The multivariate Cox regression analysis also revealed that a higher FUCA1 H-score was associated with a significantly decreased hazard of death for TNBC patients (*P = 0.003). Specifically, a one-unit increase in the FUCA1 H-score was associated with a 0.976-fold decrease in the risk of death; a ten-unit increase in the FUCA1 H-score was associated with a 22% decrease in the risk of death. These results imply that higher FUCA1 H-scores are associated with better survival outcomes after adjusting for confounding factors.

### FUCA1 expression is higher in cancer cells derived from the original breast tumor site

**Figure 3:**
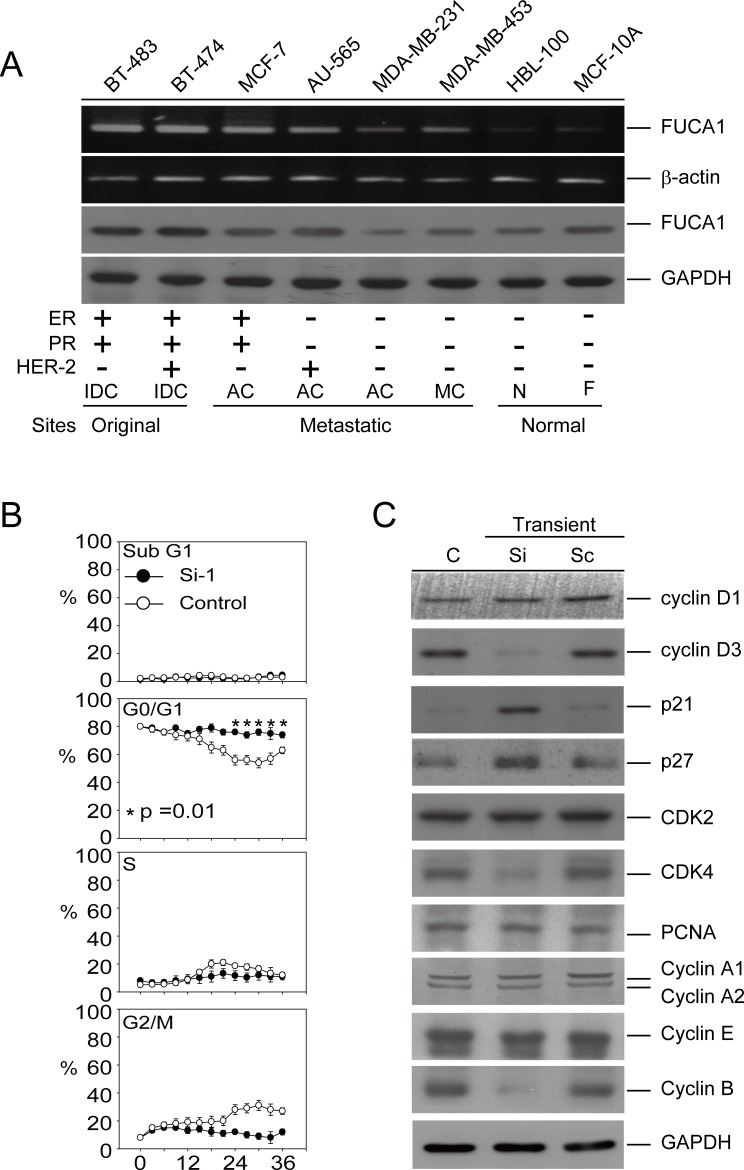
Transient inhibition of FUCA1 expression inhibits human breast cancer cell proliferation **A.** Detection of FUCA1 mRNA and protein expression by reverse transcription-PCR and immunoblotting in normal and cancerous human breast cell lines. Transformed human breast cancer cell lines: MCF-7, MDA-MB-231, AU-565, MDA-MB-453, BT-474, and BT-483. Normal human breast cell lines: MCF-10A and HBL-100. **B.** MDA-MB-231 cells were transiently transfected with short interfering RNA (siRNA) or scrambled control plasmids. Flow cytometry was performed, and the percentages of cells in G0/G1, S, and G2/M were determined using the established CellFIT DNA analysis software. Three samples were analyzed in each group, and the values represent the mean S.E. The data points represent the mean; the error bars indicate the 95% C.I. The data were analyzed using nonparametric two-sided tests (Kruskal-Wallis and Mann-Whitney tests). **C.** Immunoblot assays were performed to determine the effect of inhibiting FUCA1 protein expression on the levels of G0/G1 regulatory proteins in MDA-MB-231 cells. Protein extracts (100 μg/lane) were separated by SDS-PAGE, probed with specific antibodies, and detected by ECL.

We further explored the function of FUCA1 in human cancer cell lines. Interestingly, FUCA1 was overexpressed at the mRNA and protein levels in cancer cells isolated from the original tumor sites (BT-474 and BT-483). In contrast, FUCA1 protein expression was lower in cancer cells derived from metastatic sites (AU-565, MCF-7, MDA-MB-231, and MDA-MB-453) (Figure 3A). Interestingly, lower FUCA1 protein expression was detected in TNBC cells (MDA-MB-231 and MDA-MB-453) than in non-TNBC cells (AU-565, BT-483, BT-474, and MCF-7) (Figure 3A). These results were consistent with the clinical staging findings (Figure 1C; Table 1; *P = 0.004) and the inferior overall survival outcomes (Table 2; H-Score; *P = 0.003) [21].

**Table 2:** Univariate and Multivariate analyses for overall survival in breast cancer patients

TNBC patients (n=)		Univariate analysis			Multivariate analysis		
Parameters	Category	H.R.	95%CI	*p*-value	H.R.	95%CI	*p*-value
**Primary tumor (T)**#				0.426			0.463
	T0	0.000	0.000-0.000	0.984	0.000	0.000-0.000	0.991
	T1	0.146	0.020-1.037	0.054	0.030	0.000-1.984	0.101
	T2	0.297	0.060-1.476	0.138	0.290	0.015-5.752	0.417
	T3	0.345	0.048-2.455	0.288	0.513	0.025-10.442	0.664
	T4	1			1		
**Nodal status (N)**#				0.295			0.072
	N0	0.892	0.093-8.574	0.921	22.273	0.451-1100	0.119
	N1	1.683	0.197-14.404	0.635	51.565	1.531-1736	0.028*
	N2	3.985	0.414-38.333	0.231	0.845	0.053-13.406	0.905
	N3	1			1		
**Stage**#				0.389			0.042*
	I	0.299	0.027-3.300	0.324	1.175	0.047-29.670	0.922
	II	0.381	0.043-3.418	0.389	0.141	0.006-3.186	0.218
	III,	0.936	0.109-8.022	0.952	7.630	0.415-140.335	0.171
	IV	1			1		
**FUCA H-score (continuous)**#		0.987	0.976-0.998	0.023*	0.976	0.960-0.992	0.003*
**Non-TNBC patients (n= )**		**Univariate analysis**			**Multivariate analysis**		
**Parameters**	Category	H.R.	95%CI	p-value	H.R.	95%CI	p-value
**Primary tumor (T)**#				0.116			0.883
	T0	0.313	0.078-1.259	0.102	0.754	0.000-0.000	1.000
	T1	0.276	0.100-0.073	0.013*	0.778	0.157-3.861	0.759
	T2	0.476	0.192-1.177	0.108	0.583	0.150-2.267	0.436
	T3	0.313	0.105-0.934	0.037*	0.487	0.104-2.274	0.360
	T4	1			1		
**Nodal status (N)**#				0.186			0.775
	N0	0.536	0.236-1.216	0.136	1.733	0.317-9.477	0.526
	N1	0.454	0.194-1.063	0.069	0.830	0.198-3.479	0.799
	N2	0.536	0.261-1.099	0.089	0.789	0.255-2.434	0.679
	N3	1			1		
**Stage**#				<0.001*			0.091
	0	0.042	0.007-0.234	<0.001*	0.051	0.002-1.234	0.067
	I	0.059	0.016-0.261	<0.001*	0.000	0.000-0.000	0.975
	II	0.152	0.051-0.447	0.001*	0.039	0.004-0.383	0.005*
	III,	0.097	0.028-0.337	<0.001*	0.102	0.016-0.643	0.015
	IV	1			1		
**FUCA H-score (continuous)**#		1.002	0.995-1.008	0.639	1.003	0.995-1.011	0.436

# Cox proportional hazards model;

* Statistically significant

### Transient inhibition of FUCA1 induces G0/G1 arrest and autophagic death in MDA-MB-231 cells

Lower FUCA1 expression levels were associated with advanced-stage tumor formation with inferior survival outcomes, especially in TNBC patients (Tables 1 and 2; Figure 2D). These results suggested that tumor cells expressing lower FUCA1 protein levels should exhibit increased cell surface fucosylation, thereby enhancing the malignant capabilities of the tumor cells. A cell-based model of the negative regulation of FUCA1 in TNBC cells that expressed low FUCA1 levels (MDA-MB-231) was used to explore this hypothesis (Figure 3A). FUCA1 protein expression in MDA-MB-231 cells was transiently knocked down using siRNA, and cell cycle was analyzed by flow cytometry (Figure 3B). Transient FUCA inhibition markedly arrested MDA-MB-231 cells in G0/G1 in a time-dependent manner (Figure 3B, *P = 0.01). To explore the underlying mechanisms responsible for this effect, we transiently knocked down FUCA1 in MDA-MB-231 cells and found that the expression of G1 phase-regulated proteins, such as cyclin D3, cyclin B and CDK4, were significantly inhibited. In contrast, the expression of cyclin-dependent kinase inhibitors, such as p21/Cip1 and p27/Kip1, was increased in MDA-MB-231 cells transiently expressing FUCA1 siRNA (Figure 3C).

**Figure 4:**
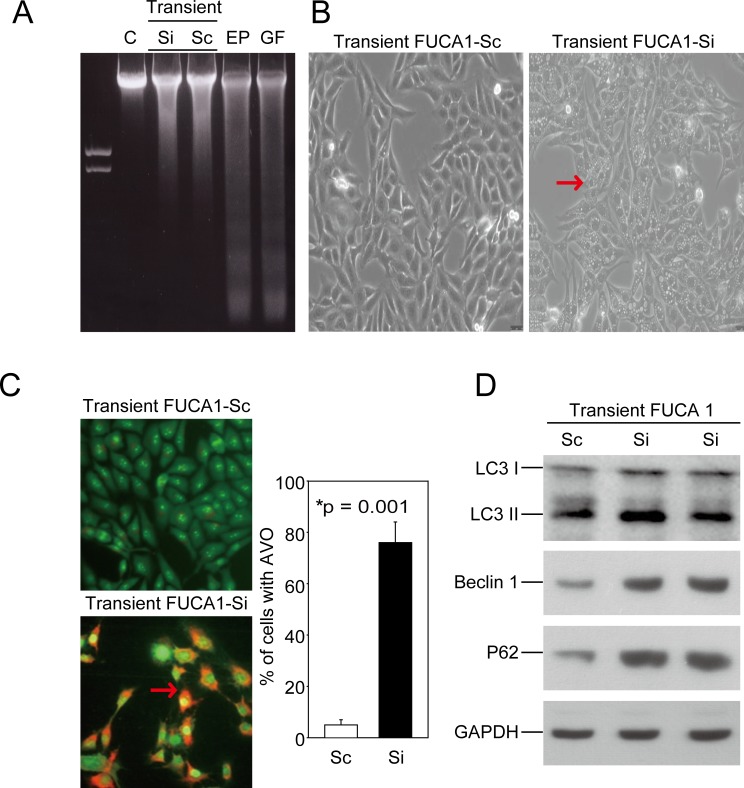
Transient inhibition of FUCA-induced autophagic cell death in MDA-MB-231 cells **A.** FUCA1 protein expression in MDA-MB-231 cells was transiently inhibited using siRNA. A DNA fragmentation assay was performed. MDA-MB-231 cells treated with Etoposide (EP, 50 μM) and griseofulvin (GF, 60 μM) were used as positive controls [48]. **B.** Cellular morphology was observed after transient transfection of the FUCA-Si plasmid. The cells appeared to lose cell-cell contact, and cytoplasmic vacuoles became apparent. **C.** Transient FUCA-knock-down cells were subjected to acridine orange staining to detect the formation of AVOs [22]. The autophagic cell death values were calculated as the percentage of acridine orange-positive cells relative to the total number of cells in each random field, and the data are presented as the mean ± SD from 3 independent experiments (*P = 0.001). **D.** MDA-MB-231 cells were transiently transfected with FUCA-Si plasmids for 24 h. Immunoblotting analyses for LC3, Beclin 1, and p62/SQSTM1 expression were performed as described in the Materials and Methods section.

Although FUCA1 inhibition induced G0/G1 arrest, it failed to cause apoptosis in MDA-MB-231 cells, as revealed by the absence of fragmented DNA in these cells (Figure 4A, lanes 2 and 3). Interestingly, transient transfecting these cells with the FUCA1-Si plasmid appeared to result in the loss of cell-cell contact; cytoplasmic vacuoles also became apparent (Figure 4B, right figure, indicated by a red arrow). A significant number of autophagic cells was observed by detecting the formation of acidic vesicular organelles (AVOs) [22], a morphological characteristic of autophagy, by acridine orange staining (Figure 4C, indicated by the red arrow). More than 75% of the cells that were transiently transfected with the FUCA1-siRNA plasmid were positive for the AVO stain (Figure 4C, *P = 0.001). The microtubule-associated protein light chain 3 (LC3) is a widely used marker for monitoring autophagy. One approach is to detect LC3 conversion (LC3-I to LC3-II) by immunoblotting because the amount of LC3-II increases as the number of autophagosomes increases [23]. Next, we analyzed autophagy in MDA-MB-231 cells after transient transfection with one of two FUCA1-siRNA plasmids (Si-1 and Si-2) (Figure 4D). There was a significant increase in endogenous LC3-II accumulation in both of the transient FUCA1-siRNA cell lines (Figure 4D). In addition, p62, or sequestosome 1 (SQSTM1), is a common component of protein aggregates that is responsible for linking polyubiquitinated proteins to the autophagic machinery [24]. p62 binds the autophagy regulator Atg8/LC3 through a region termed the LC3-interacting region [25]. The autophagy gene *beclin 1* is part of a type III PI3K complex that is required for the formation of autophagic vesicles [26]. The protein expression levels of p62 and Beclin 1 were increased in the transient FUCA1-siRNA cell lines (Figure 4D).

### Transient FUCA1 inhibition in MDA-MB-231 cells produces a selective pressure that triggers primary tumor cell metastasis

**Figure 5:**
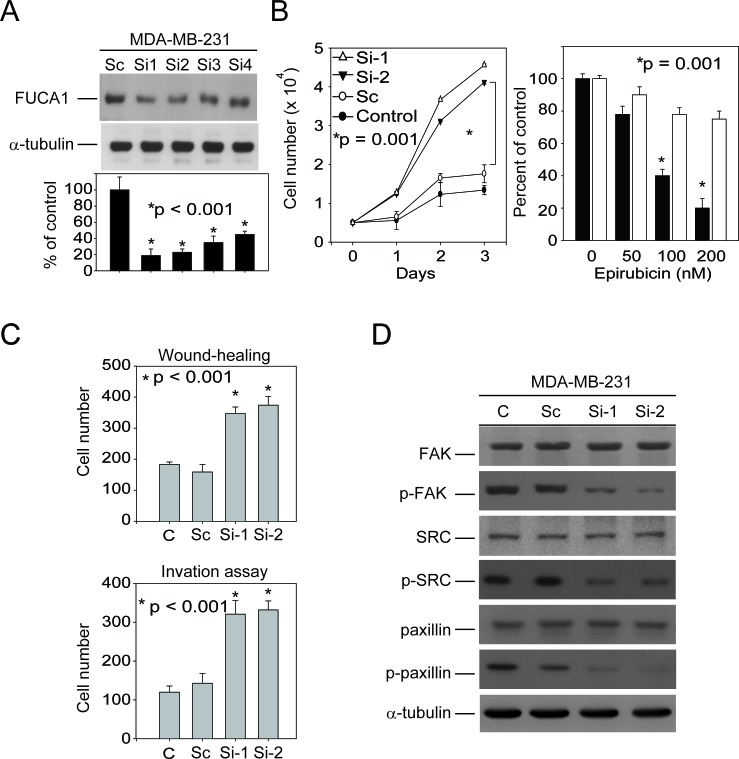
FUCA inhibition in MDA-MB-231 cells produces a selective pro-metastatic pressure on primary tumor cells **A.** Five stable FUCA1-knock-down MDA-MB-231 clones (FUCA1 Si-1 to Si-5) were selected. **B.** Cell proliferation assays were performed using wild type MDA-MB-231 cells (control) or those stably expressing FUCA1 Si-2, Si-2, or scramble (Sc) (*P = 0.001). **C.** Wound healing migration and invasion assays were performed using control MDA-MB-231 cells or those stably expressing FUCA1-Si or FUCA1-Sc for 24 h (*P < 0.001). **D.** FUCA1 expression was stably knocked down in MDA-MB-231 cells. FAK/Src protein levels in these cells were examined by immunoblotting analysis. The expression of α-tubulin was used as a control for equal protein loading.

The results presented in Figure 4B and 4C indicate that transiently transfecting the FUCA1-siRNA plasmid into MDA-MB-231 breast cancer cells induced autophagic death. However, our clinical observations indicated that FUCA1 was expressed at a lower level in advanced-stage breast cancer cells (Figure 1C, Table 1). These results suggest that the transient inhibition of FUCA1 in MDA-MB-231 cells may produce a selective metastatic pressure on the primary tumor cells. To test this hypothesis, two stable FUCA1-knock-down MDA-MB-231 clones (FUCA1-Si-1 and FUCA1-Si-2) were established for further analyses (Figure 5A). Cell proliferation assays were performed; both FUCA1-knock-down MDA-MB-231 cell lines exhibited significantly increased cell proliferation compared with the control cells (Figure 5B, *P = 0.001).

The two most commonly used anthracyclines in TNBC therapy are doxorubicin and epirubicin, which are structural analogs. Epirubicin is commonly used to treat TNBC and is usually better tolerated than doxorubicin [27]. Epirubicin can induce autophagy in MDA-MB-231 cells [28]. *In vitro* studies were performed to determine whether FUCA inhibition might cause therapeutic resistance in clinical TNBC patients. Nearly 40% of epirubicin-treated wild type MDA-MB-231 cells were viable after 48 h (200 nM epirubicin). However, FUCA1-knock-down cells were more resistant to the cytotoxicity of epirubicin (Figure 5B, right panel, *P = 0.001). These results suggest that stable FUCA1 inhibition produces a selective pressure that triggers resistance to epirubicin-induced death in MDA-MB-231 cells [28].

### Stable down-regulation of FUCA1 protein expression promotes MDA-MB-231 cell migration and invasion by inhibiting FAK/Src signaling

The stable knock-down of FUCA1 (Si-2 and Si-2) protein expression significantly enhanced cellular migration and invasion compared with wild type or FUCA-1-Sc-treated control MDA-MB-231 cells (Figure 5C, lanes 3-4 *vs*. lanes 1-2, *P < 0.001). These results suggest that FUCA1 may be capable of triggering metastasis in early-stage TNBC cancer cells.

Several recent studies have demonstrated that breast cancer epithelial cell migration is influenced by the tumor microenvironment, which includes stromal cells and the extracellular matrix (ECM) [29]. Tumor cells adhere to the ECM via integrin binding; this binding triggers the phosphorylation of two major integrin-binding proteins, FAK and Src, which are present at focal adhesions (FA), leading to FA disassembly and cell migration [30, 31]. To test whether FAK signaling is involved in FUCA1-induced cell invasion, the FAK/Src levels in MDA-MB-231 cells (Si-1 and Si-2) were examined. The levels of phosphorylated FAK (p-FAK-Tyr-576) and phosphorylated Src (p-Src-Tyr-418) were lower in FUCA1 stable knock-down cells (Figure 5D). These results suggest that FUCA facilitates invasive breast cancer cell migration and matrix adhesion via the FAK/Src signaling pathway and that this pathway may represent a potential target for preventing the metastasis of TNBC breast cancer cells induced by the down-regulation of FUCA1.

## DISCUSSION

Alpha-L-fucose is a key monosaccharide that is overexpressed during breast tumor invasion [1]. FUCA1 catalyzes the hydrolytic cleavage of terminal alpha-L-fucose residues in glycoproteins and glycolipids that are associated with the transformation to a malignant phenotype [1]. Increased fucosylation in breast cancer cells due to the stable down-regulation of FUCA1 protein expression (Figure 3) is associated with malignant transformation, invasion and metastasis. It might be expected that the enzyme FUCA1, which is involved in the breakdown of fucose-containing glycoproteins and glycolipids, would play an important role in the maintenance of the fucose content of aberrantly fucosylated glycoconjugates. Similar observations in patients with colorectal adenocarcinoma have revealed a significant decrease in FUCA1 activity in malignant tissue compared with healthy colonic mucosa in the same patient [15, 16]. These studies indicate that FUCA1 has potential utility in the diagnosis of breast cancer, particularly at stages in which the tumor has not yet disseminated [16]. In this study, the over-expression of FUCA1 (Figure 1C) was detected in early-stage breast cancer tissue from Asian patients. This phenomenon was also detected in early-stage cancer cells (BT-483 and BT-474) derived from the original tumor site. In contrast, we detected lower FUCA1 expression in TNBC (MDA-MB-231 and MDA-MB-453) cells derived from metastatic sites (Figure 3A). We also demonstrated that FUCA1 mRNA expression was higher in non-smoking patients compared with patients who smoked (Table 2, *P = 0.037). These results can be explained by our previous studies that indicated that smoking-induced breast cancer formation was associated with clinical outcomes, such as disease stage and 5-year overall survival [32, 33]. All these observations indicate that high FUCA1 expression could alter the composition and decrease the quantity of cell surface fucosylation-associated molecules, thereby limiting the invasiveness of cancer cells in early-stage breast tumors.

**Figure 6:**
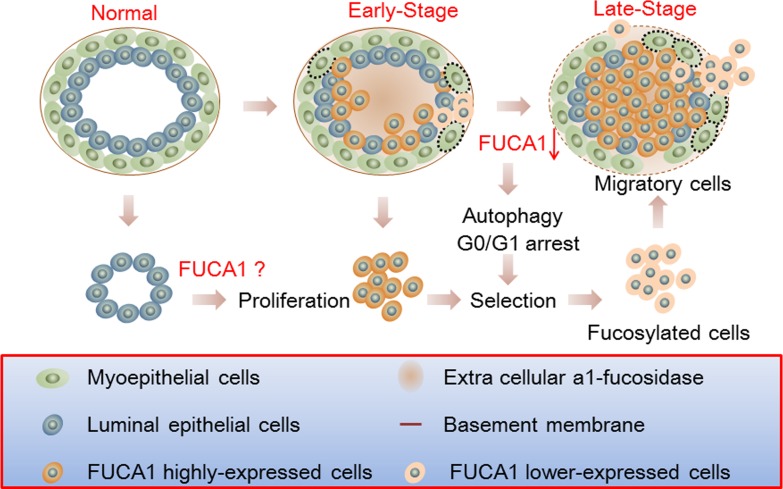
Schematic of the involvement of FUCA1 in the tumorigenic capacity of breast cancer cells Higher FUCA1 protein expression was detected in early-stage breast cancer tissues. Transient FUCA1 inhibition significantly arrested MDA-MB-231 cells in G0/G1 and induced autophagic cell death. Then, the cells with minimal levels of FUCA1 expression were selected, and these cells maintained malignant properties, such as cancer cell growth and metastasis. These effects are similar to the epithelial-to-mesenchymal transition process by which epithelial cells inhibit cell-cell interactions and reorganize the cytoskeleton to acquire mesenchymal properties and enable migration to secondary sites.

A previous study demonstrated that pretreatment with FUCA decreased the invasiveness of the highly invasive/metastatic MDA-MB-231 human breast cancer cell line [2]. The results described above imply that increased expression of FUCA1, which removes alpha-L-fucose from the tumor cell surface, may be a useful biomarker for early-stage cancer cells at the primary tumor site (Figures 1C and 3A). For example, Lewis-x antigens are overexpressed on epithelial cells of various origins, including breast cancer cells [1, 6]. Increased fucosylation of Lewis-x antigens by 5 fucosyl-transferases (FUT-3, -4, -5, -6, and -7) was detected in breast cancer cells that preferentially metastasized to the bone [20]. In this study, we found that lower FUCA1 expression was associated with highly fucosylated Lewis-x antigens, such as those detected in advanced-stage breast cancer tissues (Figures 2A and 6). To evaluate whether FUCA1 protein expression (FUCA H-score) in tumor tissue predicted overall survival, we used Cox regression proportional hazard analysis. Interestingly, the univariate Cox regression analysis revealed that FUCA H-scores were associated with the risk of death for TNBC patients (*P = 0.023) but not for non-TNBC patients (Table 2). The multivariate Cox regression analysis also revealed that higher FUCA H-scores significantly decreased the hazard for death for TNBC patients (Table 2, *P = 0.003). Therefore, FUCA H-scores significantly predicted an inferior overall survival rate for breast cancer patients in the lower expression group (*n* = 57) compared with the higher expression group (n = 102) (Figure 2D, *P = 0.009).

High FUCA expression could alter the composition and decrease the quantity of cell surface fucosylation-associated molecules, thereby limiting the invasiveness of cancer cells in early-stage breast tumors (Figure 1C and Table 1). These results suggest that tumor cells expressing lower FUCA protein levels should exhibit increased cell surface fucosylation, which would enhance the malignant potential of the tumor cells. To explore this hypothesis, FUCA1 was knocked down using siRNA in MDA-MB-231 cells, which express low levels of FUCA1, to create a cell-based research model [34, 35]. Transient FUCA1 inhibition markedly arrested MDA-MB-231 cells at G0/G1 (Figure 3B). Furthermore, transient FUCA inhibition resulted in the loss of cell-cell contact; cytoplasmic AVO vacuoles, which represent a morphological characteristic of autophagy, also became apparent [22]. In contrast, we detected increased proliferation, invasion and migration in established stable FUCA1 knock-down (Si-1 and Si-2) cell lines. These observations indicated that transient FUCA inhibition in early-stage breast cancer cells may trigger a selective pressure to generate metastatic primary tumor cells (Figure 6). Then, cells with minimal FUCA1 expression are selected for the maintenance of malignant properties, such as growth and metastasis.

## MATERIALS AND METHODS

### Reagents

The following drugs were used: epirubicin (EPI; Pharmaceutical Partners of Canada Inc.), doxorubicin (Pfizer Canada Inc.), and chloroquine (Sigma-Aldrich).

### Cell lines

We selected several breast cancer cell lines that were isolated from primary invasive ductal breast carcinomas (IDC; BT-483 (HTB-121) and BT-474 (HTB-20), American Type Culture Collection, ATCC, Manassas, VA) [36], metastatic pleural or pericardiac effusions (AC; MCF-7 (HTB-22), MDA-MB-453 (HTB-131), and AU-565 (CRL-2351), ATCC, Manassas, VA) [37-39], or other metastatic deposits (MC; MDA-MB-231 (HTB-26), ATCC, Manassas, VA) [40]. Additionally, a continuous cell line (HBL-100, denoted as N; Cat. No. HTB-124; ATCC, Manassas, VA) obtained from a primary culture of cells derived from an early lactation sample of human milk and a normal human breast epithelial cell line (MCF-10A, denoted as F; Cat. No. CRL-10317; ATCC, Manassas, VA) were used as controls. MCF-10A cells were maintained in complete MCF-10A culture medium composed of a 1:1 mixture of Dulbecco's modified Eagle's medium (DMEM) and Ham's F12 medium supplemented with 100 ng/mL cholera enterotoxin, 10 μg/mL insulin, 0.5 μg/mL hydrocortisol, and 20 ng/mL epidermal growth factor (Life Technologies, Rockville, MD, USA). MCF-7, MDA-MB-231, HBL-100, and MDA-MB-453 cells were maintained in DMEM; AU-565, BT-474 and BT-483 cells were cultured in RPMI-1640.

### Cell proliferation and viability assays

Cell growth and proliferation were determined using the 3-(4,5-dimethylthiazol-2-yl)-2,5-diphenyltetrazolium (MTT) assay [41]. This assay was repeated four times with duplicate samples.

### RNA interference

FUCA1 expression was ablated in MDA-MB-231 breast cancer cells using at least two independent siRNA clones. Scrambled sequences of each siRNA were used as controls (Supplementary file 1). After Basic Local Alignment Search Tool (BLAST) analysis to verify the absence of significant sequence homology with other human genes, the selected sequences were inserted into the pSUPER vector (OligoEngine Co., Seattle, WA, USA) digested with *Bgl*II and *Hin*dIII to generate the pSUPER-FUCA1-Si and pSUPER-scramble vectors. The sequences of all the constructs were confirmed by DNA sequencing. The transfection protocol has been described previously [33]. Briefly, 1.5 × 10^5^ cells were washed twice with phosphate-buffered saline and mixed with 0.5 μg of plasmid. One pulse was applied for 20 msec under a fixed voltage of 1.2 kV on the pipette-type microporator MP-100 (Digital Bio, Seoul, Korea).

### Generation of stable FUCA1-siRNA-expressing cell lines

At least three clones of MDA-MB-231 cells that stably expressed FUCA1 siRNA or scrambled control siRNA were generated. All the experiments were performed using multiple sub-clones of each cell line with reproducible results. The pSUPER-FUCA1-Si and pSUPER-scramble vectors were transfected into the cells, and stable integrants were selected 72 h later with G418 (4 mg/mL). After 30 days in selective medium, two G418-resistant clones (FUCA1-Si-1 and FUCA-Si-2) were isolated; these clones demonstrated a > 80% reduction in mRNA and protein expression compared with the control clones (scramble control: FUCA1-Sc).

### Protein extraction, western blotting, and antibodies

To examine protein expression, human breast tumor cells were thawed in lysis buffer that contained protease inhibitors. Protein extracts (50 μg) from each sample were separated by 12% SDS-polyacrylamide gel electrophoresis, transferred, and analyzed by Western blotting. Primary antibodies were purchased from multiple sources: anti-FUCA1, Abnova Corporation; anti-p-Src418, Cat. No. E011091, Enogene; anti-Src, Cat. No. ab16885, clone 327, Abcam, Cambridge, MA, USA; anti-p-FAK576, Cat. No. sc-16563, clone Tyr576, Santa Cruz Biotechnology, CA, USA; p-FAK576, anti-FAK, Cat. No. sc-1688, clone H-1, Santa Cruz Biotechnology, Santa Cruz, CA, USA; anti-Paxillin, Cat. No. sc-5574, clone H114, Santa Cruz Biotechnology, Santa Cruz, CA, USA; anti-p-paxillin Tyr-118, Cat. No. sc-101774, clone Tyr-118, Santa Cruz Biotechnology, Santa Cruz, CA, USA; anti-GAPDH, Cat. No. sc-32233, clone 6c5, Santa Cruz Biotechnology, Santa Cruz, CA, USA; anti-p62/SQSTM1, PM045, MBL, Nagoya, Japan; and anti-Beclin 1 (3738), Cell Signaling Technology, Ipswich, MA, USA. Secondary antibodies (alkaline phosphatase-coupled anti-mouse and anti-rabbit IgG) were purchased from Santa Cruz Biotechnology (Santa Cruz, CA, USA). GAPDH expression was used as a control for equal protein loading.

### Flow cytometry analysis of cell cycle

MDA-MB-231 cells were synchronized as previously described [42]. After the cells reached 70-80% confluency, they were rendered quiescent by incubation for 24 h in RPMI 1640 containing 0.04% fetal calf serum (FCS) and then challenged with 10% FCS. After the cells were released using trypsin-EDTA, they were harvested at various time points, washed twice with PBS/0.1% dextrose, and fixed in 70% ethanol at 4°C. Nuclear DNA was stained using a reagent containing propidium iodide (50 μg/mL) and DNase-free RNase (2 U/mL) and measured using a fluorescence-activated cell sorter. The populations of nuclei in each phase of the cell cycle were determined using the established CellFIT DNA analysis software (Becton Dickenson, San Jose, CA).

### Analysis of DNA fragmentation

DNA fragmentation was analyzed as previously described [43]. Briefly, cells were seeded in 100-mm dishes. DNA was extracted twice with equal volumes of phenol and then once with chloroform-isoamyl alcohol (24:1, v:v). DNA was precipitated with 0.1 volumes of sodium acetate, pH 4.8, and 2.5 volumes of ethanol at −20°C overnight, followed by centrifugation at 13,000 x g for 1 h. Genomic DNA was quantified, and equal amounts of DNA from each sample were electrophoresed in a 2% agarose gel. DNA was visualized by ethidium bromide staining.

### Cell staining with acridine orange to detect autophagy

Cell staining with acridine orange (Sigma Chemical Co.) was performed according to published procedures [44, 45]. A final concentration of 1 mg/mL acridine orange was added to the cells for 20 min. Photographs were obtained with a fluorescence microscope (Leica DMI4000B, Wetzlar Germany) equipped with a 100-W mercury lamp, a 490-nm band-pass blue excitation filter, a 500-nm dichroic mirror, and a 515-nm long-pass barrier filter.

### Wound healing cell migration assay

MDA-MB-231 cells expressing FUCA1-Si-1, FUCA1-Si-2, or FUCA1-Sc were seeded into 6-well plates and allowed to reach 70% confluency in complete medium. The cell monolayers were wounded with a plastic pipette tip (1 mm in diameter). The wounded monolayers were washed several times with PBS to remove cell debris and were incubated in medium for an additional 24 h. Cell migration into the wound area and the average number of migrating cells were determined using an inverted microscope at various time points.

### *In vitro* invasion assays

*In vitro* invasion assays were performed using 10-mm Transwell chambers that contained Matrigel-coated polycarbonate membranes with 8-μm pores (Corning Costar, Cambridge, MA, USA) as previously described [46]. MDA-MB-231 cells expressing the FUCA-Si-1, FUCA1-Si-2 or FUCA1-Sc plasmids were trypsinized and suspended at a final concentration of 5 × 10^5^ cells/mL in serum-free L15 medium. The cell suspensions were added to the upper Transwell chambers. The bottom chambers contained medium with 5% FBS as a chemoattractant. After a 24-h incubation at 37°C in 5% CO_2_ and 95% air, all the non-invading cells were removed from the upper surface of the Transwell membrane with a cotton swab. The invading cells were fixed with 100% methanol, stained with hematoxylin and eosin (Nanjing Sunshine Biotechnology Ltd., Nanjing, China), and counted under a microscope. Ten fields were counted for each assay.

### Patient samples

The participants (*n* = 236) in this study provided their written informed consent. The study and consent procedures were approved by the Research Ethics Committee of Taipei Medical University Hospital. Pairs of human breast tumor and adjacent normal epithelial tissues were obtained from anonymous donors according to a protocol that had been approved by the Institutional Review Board (TMU-JIRB, No. 201407014). All the clinical investigations were conducted according to the principles expressed in the Declaration of Helsinki. Histological inspections revealed that all the patient samples consisted of more than 80% tumor tissue. All the samples (each paired tumor *vs*. normal tissue) were collected and categorized according to their clinical characteristics.

### Laser capture microdissection (LCM)

Frozen sections from the breast tumor samples were prepared for LCM [33]. In this study, we collected tissues from tumors of different stages (stages 0-4, *n* = 12). Sections that had been stained with HistoGene (Cat. No. KIT0401; Arcturus Engineering, Mountain View, CA, USA) were subjected to LCM using a PixCell IIe system (Arcturus Engineering, Mountain View, CA, USA) [47]. The parameters for LCM included a laser diameter of 8 μm and a laser power of 48–65 mW. A total of 15,000 laser pulse discharges were used for each specimen to capture ∼10,000 morphologically normal epithelial cells or malignant carcinoma cells for each case. Each population was visualized under a microscope to ensure that the captured cells were homogeneous. Then, the caps with the captured cells were fitted onto 0.5-mL Eppendorf tubes containing 42 μL of lysis buffer. RNA was isolated by following the standard protocol of the PicoPure RNA Isolation Kit (Cat. No. KIT0204; Arcturus Bioscience, Mountain View, CA). The purified RNA was analyzed by reverse transcription and real-time quantitative PCR.

### RNA isolation and real-time quantitative PCR

Total RNA from human breast tumors and normal tissue samples that had been acquired directly from patients was isolated using Trizol (Invitrogen, Carlsbad, CA, USA) according to the manufacturer's protocol. The FUCA1-specific PCR primers were synthesized as F-CGCAGAGTTTGCTTGGAC and R-GGTGGAGAAGAGAAGTTCGT, and the β-glucuronidase (GUS)-specific primers were synthesized as F-AGTGTTCCCTGCTAGAATAGATG and R-AAACAGCCCGTTTACTTGAG. A LightCycler thermocycler (Roche Molecular Biochemicals, Mannheim, Germany) was used for real-time quantitative PCR. The FUCA1 mRNA fluorescence intensity was measured and normalized to GUS expression using the built-in software (Roche LightCycler Version 4) [33].

### Antibodies, immunohistochemistry and microscopic scoring

The localization of FUCA1 and fucosylated Lewis-x antigen proteins in breast tumor tissues was detected by immunohistochemistry. Paraffin-embedded breast tumor tissues excised from patients were cut into 5-μm slides. The sections were preincubated in 3% H_2_O_2_ and 0.3% Triton X-100 before microwaving for antigen retrieval. The sections were microwaved in Tris buffer (pH 6) for 10 min. Then, the sections were blocked in 5% horse serum (Chemicon, Temecula, CA, USA) for 30 min and subsequently incubated with antibodies targeting FUCA1 (Cat. No. H00002517-M01, clone 1D4; Abnova) or anti-fucosylated Lewis-x monoclonal antibody HECA-452 (BD Biosciences, MD, USA) [6, 20] diluted 1:100 for 2 h at room temperature. After incubation with the primary antibodies, the sections were stained according to the streptavidin-biotin-peroxidase method using an LSAB 2 kit (DAKO, Carpinteria, CA, USA). Briefly, the sections were washed in phosphate-buffered saline and incubated with biotinylated anti-rabbit secondary antibodies. Then, the samples were washed again in the same buffer and incubated with the streptavidin-biotin-peroxidase complex. Staining was complete after incubation with the substrate-chromogen solution. The duration of the incubation in the DAB solution was determined by low-power microscopic inspection. Then, the slides were washed, dehydrated and coverslipped using DPX (Sigma-Aldrich, St. Louis, MO, USA). Adjacent sections and slides were counterstained with hematoxylin for general histological evaluation.

Immunoexpression was scored by two pathologists (C.H.W. and S.H.T.) using a multi-headed microscope to obtain a consensus for each sample. Staining was evaluated based on a combination of both the percentage and the intensity of the positively stained tumor cells to generate an H-score, which was calculated using the following equation: H-score = AC*Pi*(*i*+1), where *i* is the intensity of the stained tumor cells (0 to 4+), and *Pi* is the percentage of stained tumor cells for each intensity.

### Statistical methods

All the data are expressed as the mean of at least three experiments with 95% confidence intervals (CIs), unless otherwise stated. A paired t-test was used to compare FUCA1 mRNA expression in paired normal *vs*. tumor tissues from breast cancer patients. The fold ratios of FUCA1 mRNA expression in tumors *vs*. normal samples were compared using the Mann-Whitney U test. Pearson correlation coefficient tests were used to identify associations between FUCA1 protein expression and clinicopathological variables and Lewis expression levels. The endpoint was overall survival, which was calculated from the starting date of surgery to the event date. The median follow-up was 60 months (range, 2-152 months). Survival analyses were performed using the Cox proportional hazards model. Survival curves were plotted using the Kaplan-Meier method, and log-rank tests were performed to evaluate prognostic differences between groups for categorical variables. All the statistical comparisons were performed using SigmaPlot graphing software (San Jose, CA, USA) and Statistical Package for the Social Sciences, v. 16.0 (SPSS, Chicago, IL, USA). All the statistical tests were two-sided. A p-value of 0.05 or less was considered statistically significant.

## SUPPLEMENTARY MATERIAL TABLES


