# Supplementary material for: Down-regulation of α-L-fucosidase 1 expression confers inferior survival for triple-negative breast cancer patients by modulating the glycosylation status of the tumor cell surface
Source: Oncotarget. 2015 May 22;6(25):21283–300. doi: 10.18632/oncotarget.4238 (PMC4673265; doi:10.18632/oncotarget.4238)
Supplement: Supplementary file 1 [file oncotarget-06-21283-s001.pdf]

Down-regulation of α-L-fucosidase 1 expression confers inferior survival for triple-negative breast cancer patients by modulating the glycosylation status of the tumor cell surface

Supplementary Material

Additional file 1:

No.	Name	Sequences (5' to 3')	Quantity	Quality
**1.**	FUCA1 siF4 SC F	GAT CCC CCG ATA CAC TAA CAA CAC TAT TCA A GA GAT AGT GTT GTT AGT GTA TCG TTT TTA	**2** O.D.	OPC
**2.**	FUCA1 siF4 SC R	AGC TTA AAA ACG ATA CAC TAA CAA CAC TAT CT C TTG AAT AGT GTT GTT AGT GTA TCG GGG	**2** O.D.	OPC

No.	Name	Sequences (5' to 3')	Quantity	Quality
**1.**	FUCA1siF4 F	GAT CCC CCC TCA ACT ACA AAG ATA ACT TCA AG A GAG TTA TCT TTG TAG TTG AGG TTT TTA	**2** O.D.	OPC
**2.**	FUCA1siF4 R	AGC TTA AAA ACC TCA ACT ACA AAG ATA ACT CT C TTG AAG TTA TCT TTG TAG TTG AGG GGG	**2** O.D.	OPC

No.	Name	Sequences (5' to 3')	Quantity	Quality
1.	FUCA1 siRNA1 Forward	GAT CCC CAC AAG TGG GAG ATG TGC ACT TCA AGA GAG TGC ACA TCT CCC ACT TGT T TT TTA	2 O.D.	OPC
2.	FUCA1 siRNA1 Reverse	AGC TTA AAA AAC AAG TGG GAG ATG TGC A CT CTC TTG AAG TGC ACA TCT CCC ACT TG T GGG	2 O.D.	OPC
3.	FUCA1 siRNA2 Forward	GAT CCC CCC GCA GTA CCA GCG CTT CAT TCA AGA GAT GAA GCG CTG GTA CTG CGG TTT TTA	2 O.D.	OPC
4.	FUCA1 siRNA2 Reverse	AGC TTA AAA ACC GCA GTA CCA GCG CTT C AT CTC TTG AAT GAA GCG CTG GTA CTG CG G GGG	2 O.D.	OPC
5.	FUCA1 sc Forward	GAT CCC CGA GGA GAA TGT GCA CAT GCT TCA AGA GAG CAT GTG CAC ATT CTC CTC T TT TTA	2 O.D.	OPC
6.	FUCA1 sc Reverse	AGC TTA AAA AGA GGA GAA TGT GCA CAT G CT CTC TTG AAG CAT GTG CAC ATT CTC CT C GGG	2 O.D.	OPC
